# Gastrodin alleviates bone damage by modulating protein expression and tissue redox state

**DOI:** 10.1002/2211-5463.12991

**Published:** 2020-10-21

**Authors:** Bowen Zheng, Chunling Shi, Fenik K. Muhammed, Jia He, Adil O. Abdullah, Yi Liu

**Affiliations:** ^1^ School and Hospital of Stomatology Liaoning Provincial Key Laboratory of Oral Diseases China Medical University Shenyang China; ^2^ Present address: Orthodontics Department Erbil Polytechnic University Kurdistan Region Erbil Iraq; ^3^ Present address: Restorative Department Erbil Polytechnic University Kurdistan Region Erbil Iraq

**Keywords:** apoptosis, fluorosis, gastrodin, oxidative stress, skeletal fluorosis

## Abstract

Fluorosis is a common disease characterized by disruptions in bone metabolism and enamel development. The production of reactive oxygen species is thought to play an important role in fluorosis. Gastrodin (4‐hydroxybenzylalcohol4‐O‐beta‐d‐glucopyranoside) has been reported to have antioxidative activity, and so here we examined whether gastrodin has protective effects against oxidative stress and bone tissue toxicity in rats with fluorosis. Wistar rats were given different doses of gastrodin 1 month after fluoride administration, and samples of blood, bone and teeth were collected after 2, 3 and 4 months; glutathione peroxidase glu, CAT and SOD levels in the fluorosis group were lower than those in the control group. Gastrodin treatment in rats ameliorated oxidative stress and fluoride accumulation that were induced by fluoride; treatment with 400 mg·kg^−1^ gastrodin protected trabecular bone structure and reduced femur and alveolar bone injury in rats with fluorosis. Enhanced expression of cysteinyl aspartate‐specific proteinase (caspase) 3, caspase‐9 and Bax and decreased expression of Bcl‐2 induced by fluoride were also reversed by gastrodin. In summary, the present data suggest that gastrodin, and in particular a dose of 400 mg·kg^−1^, can improve the antioxidative capacity of rats, reduce concentration of fluoride in tissues, alleviate bone damage and modulate expression of Bcl‐2, Bax, caspase‐3 and caspase‐9.

## Graphical abstract

Here, we show that gastrodin, which is extracted from Gastrodia and has anti‐cell death, antiapoptosis and antioxidative activities, can reverse fluoride‐induced bone injury and apoptosis in a dose‐dependent way. This protective effect of gastrodin is likely to be exerted via inhibiting oxidative stress. Therefore, gastrodin may have potential for future development into a possible treatment for fluorosis.

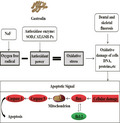

**Abbreviations**
ALP: alkaline phosphatase
caspase: cysteinyl aspartate‐specific proteinase
CAT: catalase
GSH‐Px: glutathione peroxidase glu
H&E: hematoxylin and eosin
MDA: malondialdehyde
NAC: N‐acetyl‐L‐cysteine
NaF: sodium fluoride
ROS: reactive oxygen species
SD: standard deviation
SOD: superoxide dismutase
TRAP: tartrate‐resistant acid phosphatase

Fluorosis is a common disease characterized by disruptions in bone metabolism and enamel development that results in skeletal fluorosis and dental fluorosis, respectively [1]. Exposure to fluoride leads to toxicity to various soft tissues, such as nephrotoxicity [2], cardiovascular system toxicity [3], thyroid toxicity [4], among others. Clinical research has also found that skeletal fluorosis may cause bone pain, stiffness, and spinal and limb deformities in pathology, and an increase in bone mass is observed in animal models with a concomitant reduction in ultimate load and stiffness [5]. The production of reactive oxygen species (ROS) is thought to play an important role in the toxic effects of fluorosis [6]. Oxidative stress occurs when the production of ROS in the system exceeds the ability of the system to neutralize and eliminate them. Researchers [7] reported the increase in malondialdehyde (MDA), ascorbic acid, glutathione peroxidase and glutathione levels in children aged 3–10 years with endemic fluorosis, and also oxidative stress increased. Li and Ca [8] revealed that human superoxide dismutase (SOD) was reduced in areas with endemic fluorosis. In addition, studies have shown that oxidative stress activates apoptotic signaling pathways *in vivo* and *in vitro* [9].

Cysteinyl aspartate‐specific proteinase (caspase) is the main enzyme for cell death and is sensitive to ROS [10]. Moreover, studies have suggested that sodium fluoride (NaF) induces excessive production of ROS, and thus will reduce their activity and the life span of osteoblasts in bone, resulting in reduced osteoblast formation, and the use of antioxidants has been observed to eliminate osteoblast apoptosis [11].

Increased levels of ROS lead to oxidative stress and apoptosis, and there is strong evidence that antioxidants [12] prevent the oxidative stress and apoptosis of tissues caused by the excessive fluoride. Therefore, natural substances, especially Chinese herbal medicines with antioxidant properties, may regulate bone cell activity and maintain bone structure.

Gastrodin (4‐hydroxybenzylalcohol4‐O‐beta‐d‐glucopyranoside) is the main bioactive component of the traditional Chinese medicine Gastrodia elata officially listed in the Chinese Pharmacopoeia [13]. Previous studies show that gastrodin is involved in some pharmacological activities, such as relaxation on smooth muscle [14], anti‐cell death, anti‐apoptosis [15] and anti‐oxidative activities [16]. It is an effective antioxidant and free radical scavenger that reduces lipid peroxidation levels, inhibits unconjugated oxidative phosphorylation [17] and increases expression of genes encoding antioxidant proteins [18]. Recently, Huang *et al*. [19] revealed that gastrodin has a protective effect on osteoporosis through the reduction of ROS. The detection of active oxygen production, MDA and bone SOD activity revealed that gastrodin could inhibit glutamate‐induced oxidative damage[20]. In addition, quite a few scholars have found the effects of gastrodin against hydrogen peroxide‐induced oxidative stress, apoptosis and death in RAW264.7 cells, which is a kind of osteoclast precursor cell [21]. Therefore, it may be a broad‐spectrum antioxidant and free radical scavenger that is effective against most target organs of the body, maintaining the redox balance *in vivo*.

Considering the pharmacological properties and potential advantages of the traditional Chinese medicine gastrodin for anti‐inflammatory, antioxidative and antiapoptotic treatment of diseases, and that the effect of gastrodin on oxidative stress and bone tissue toxicity of fluorosis were poorly understood, it is proposed to explore the potential antifluorosis effect of gastrodin and its feasible mechanism.

## Materials and methods

### Materials and reagents

Gastrodin was purchased from Chengdu Herbpurify CO., LTD (Chengdu, China). NaF was from Sinopharm Chemical Reagent CO., LTD (Shanghai, China). Sodium chloride injection (0.9%) was obtained from Shenyang North Pharmaceutical Factory (Shenyang, China). Ether and medical anhydrous EtOH (ethanol) were purchased from Beijing Chemical Works (Beijing, China). The antibody against Bax, caspase‐3 and caspase‐9 was from Proteintech Co. Ltd (Wuhan, China). The antibody to Bcl‐2 was purchased from Santa Cruz Biotechnology (Santa Cruz, CA, USA). The Catalase Assay Kit was from Nanjing Jiancheng Bioengineering Institute (Nanjing, China). Other chemicals were provided by Beyotime Biotechnology Co. (Jiangsu, China). Mice MC3T3‐E1 cells (ATCC, Manassas, VA, USA) were grown in alpha‐MEM media (Invitrogen, Carlsbad, CA, USA) containing 10% FBS, 1% penicillin and streptomycin (Sigma, Taufkirchen, Germany).

### Animals and gastrodin supplementation

**Fig. 1:**
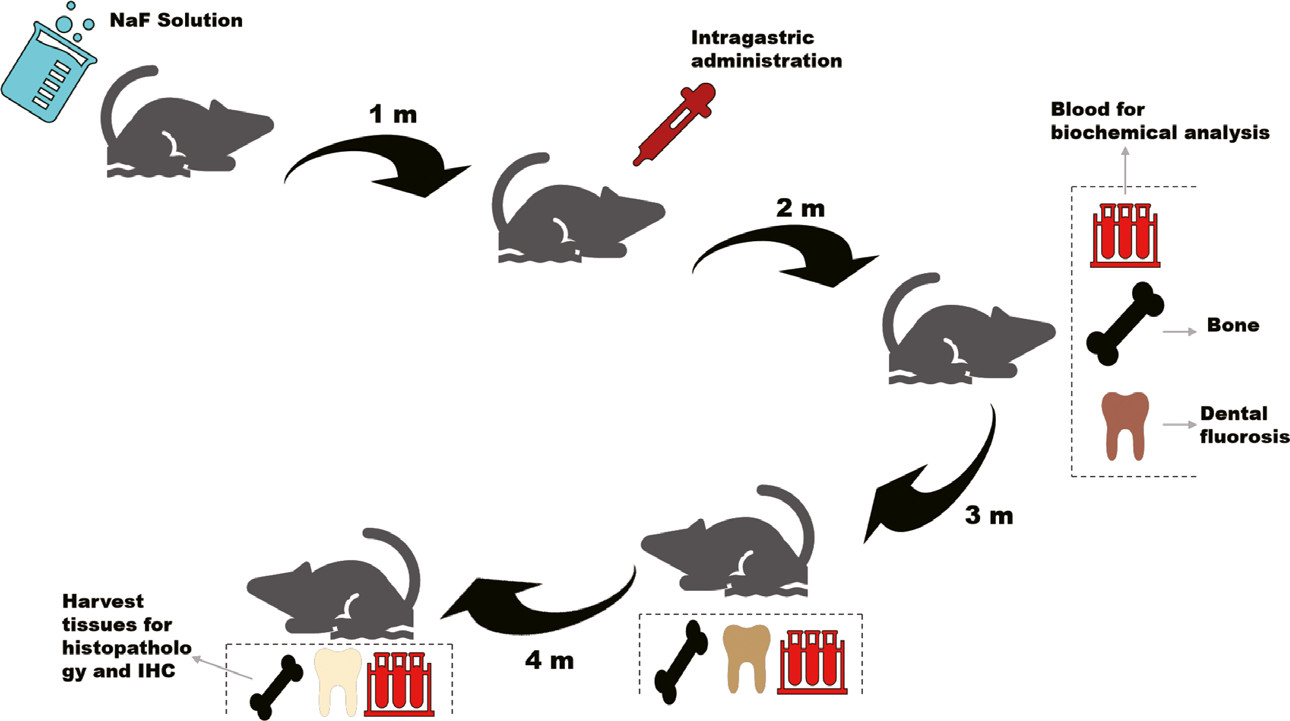
The animal experiment flowchart. IHC, immunohistochemistry.

The experiment was approved by the Ethical Committee and the Laboratory Animal Center of China Medical University, Shenyang, Liaoning, China. A total of 72 five‐week‐old Wistar male rats, average weight 180.95 g, were purchased from the Liaoning Changsheng Biotechnology Co., Ltd. (Benxi, Liaoning Province, China). There was no significant difference in the initial body weights of the rats. After a week of balanced feeding, rats were randomly divided into six groups respectively, and each group contained 12 animals: control group (basal feed, distilled water), gastrodin control group (basal feed, distilled water, 400 mg·kg^−1^ gastrodin), fluorosis group (basal feed, 150 mg·L^−1^ NaF water), low‐dose gastrodin group (basal feed, 150 mg·L^−1^ NaF water, 100 mg·kg^−1^ gastrodin), medium‐dose gastrodin group (basal feed, 150 mg·L^−1^ NaF water, 200 mg·kg^−1^ gastrodin) and high‐dose gastrodin group (basal feed, 150 mg·L^−1^ NaF water, 400 mg·kg^−1^ gastrodin). Feed was purchased from Shenyang Yuhong Qianmin animal experimental feed factory, and fluorine content in feed was less than or equal to 0.1 mg·kg^−1^. Distilled water with NaF was administrated to the rats (*n* = 48) for 4 months (150 mg·L^−1^, orally). From the second month, gastrodin was given to rats that were dissolved in distilled water and freshly prepared before administration every day. Distilled water alone was intragastric administration into the control group. The weight of each group and the occurrence of dental fluorosis were recorded before administrating fluoride (T0) and at 1 month (T1), 2 months (T2), 3 months (T3) and 4 months (T4). Rats were euthanized under overdosed anesthesia. The animal experiment flowchart is manifested in Fig. 1.

At T2, T3 and T4, four rats in each group were randomly selected, weighed and sacrificed with overdose ether anesthesia (Beijing Chemical Plant, Beijing, China); blood and teeth were collected; and serum samples of rats were obtained by centrifugation (3000 ***g***, 4 °C; Eppendorf centrifuge 5430 r, Eppendorf Co., Shanghai, China) for 10 min and frozen at −80 °C until use. The bone was promptly removed. A part of the bone was fixed, and the remaining tissues were stored at −80 °C until use. Serum Ca and MDA concentration, the activity of serum glutathione peroxidase glu (GSH‐Px), catalase (CAT), tartrate‐resistant acid phosphatase (TRAP) and alkaline phosphatase (ALP) of the serum, the activity of SOD of the bone and the fluoride concentration of bone and teeth were observed. At T4, hematoxylin and eosin (H&E) staining and immunohistochemical staining of bone were performed to observe the pathological changes of bone tissue.

### Fluoride measurements

Bone samples were collected and cleaned by removing the soft tissues, splitting open and removing all marrow with cold PBS. The bone and tooth specimens were dried for 4 h at 105 °C before ashing; the bone was heated in a smoke‐free environment in a fume hood, then ashed for 6 h at 550 °C, dissolved in 1N HCl and neutralized by the addition of 1N NaOH. A standard method with the ion‐specific fluoride‐electrode (Shanghai Weiye Instrument Plant, Shanghai, China) was used to determine the fluoride concentration of each dissolvent.

### Biochemical assay

The measurement in whole blood samples was determined using related kits according to the manufacturer’s instructions. The SOD concentration in the bone was determined using Total SOD Assay Kit with WST‐8 according to the protocol.

### Bone histomorphometry

After fixation using 4% paraformaldehyde for 24 h, the bone was transferred to ethylene diamine tetraacetic acid decalcifying fluid (Solarbio, Beijing, China) for decalcification for 30 days; histological sections of 5 μm thickness were prepared and stained with H&E and immunohistochemical experiment. The permanent slides were then examined under the light microscope.

### Immunohistochemical staining

The sections were treated with 0.3% H_2_O_2_ in EtOH to inactivate endogenous peroxidase. The sections were digested with 0.2% Triton, blocked with 5% BSA (SABC kit; Wuhan Boster Biological Technology, Ltd., Wuhan, China), incubated for 2 h with a 1 : 50 dilution of anti‐rabbit caspase‐3, caspase‐9, Bax antibody (Protein Tech Group, Wuhan, China) and anti‐mouse Bcl‐2 Ig (Santa Cruz Biotechnology) at 37 °C, and treated with DAB chromogenic liquid (SABC kit). The sections were counterstained with hematoxylin. Then the images were captured by a light microscopy (Nikon Eclipse 80I; NIKON, Tokyo, Japan). Eight positive expression areas were selected from each field, and the average absorbance was measured using an image analysis system. Strong expression means low absorbance and vice versa.

### Western blot analysis

Total protein of bone was extracted using the tissue protein extraction kit based on the manufacturer's instruction. The cells were removed from the culture plates, resuspended in PBS and then collected via centrifugation. Cell lysis was done using lysis buffer at 4 °C for 20 min. To eliminate cell debris, we sonicated the cells and then extracted them via 30‐minute high‐speed centrifugation at 4 °C. Protein assay system (Bio‐Rad, Hercules, CA, USA) was used to evaluate the concentration of the protein. Subsequently, the proteins (50 mg) were resolved on SDS/PAGE and then transferred onto nitrocellulose membranes. Next, the membranes were incubated together with antibodies specific for the desired proteins. The proteins were detected using an enhanced chemiluminescence western blotting kit (Pierce, Rockford, IL, USA) as per the manufacturer's protocol.

### Statistical analysis

Statistical analyses were performed using spss 22.0 for windows (SPSS Inc., Chicago, IL, USA). All data were expressed as the mean ± standard deviation (SD). One‐way ANOVA with Bonferroni's test was used for the determination of differences in measurements between groups for multiple comparisons, and *P* < 0.05 was considered significant.

## Results

### Effect of gastrodin on the growth of rats with fluorosis

**Fig. 2:**
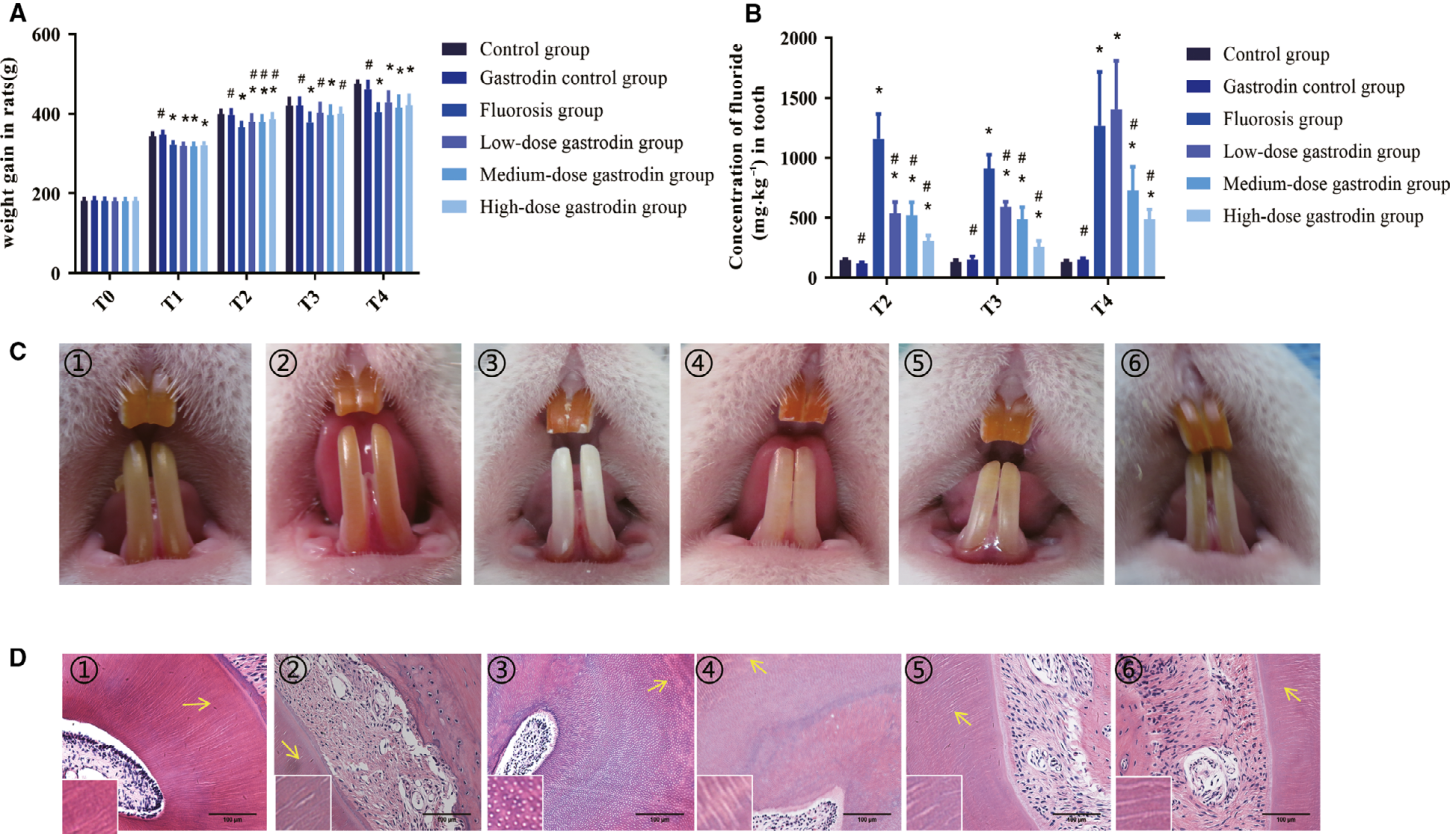
Gastrodin improves the growth and development of fluorosis in rats. (A) Rat general weight status. (B) Dental fluoride concentration among six groups. (C) Dental fluorosis of rats in each group. (D) The dentin morphology of each group during the experiment. (A) T0, *n* = 12; T1, *n* = 12; T2, *n* = 12; T3, *n* = 8; T4, *n* = 4. (B) *n* = 4. (D) Original magnification, ×200; scale bars: 100 μm. 1: The control group; 2: the gastrodin control group; 3: F; 4: fluoride + gastrodin (100 mg·kg^−1^); 5: fluoride + gastrodin (200 mg·kg^−1^); 6: fluoride + gastrodin (400 mg·kg^−1^). Data are expressed as the mean ± SD (*n* = 4). Data were analyzed by one‐way ANOVA with Bonferroni's test for multiple comparisons (A, B). *P < 0.05 versus control group; ^#^ *P* < 0.05 versus fluorosis group; **P < 0.01 versus control group; ^##^ *P* < 0.01 versus fluorosis group;***P < 0.001 versus control group; ^###^ *P* < 0.001 versus fluorosis group.

First, we measured the weight gain of each group of rats and observed the effect of gastrodin. Data in Fig. 2A show that gastrodin (100, 200 and 400 mg·kg^−1^) could ameliorate the growth of fluorosis in rats, suggesting that gastrodin partially inhibited fluoride‐induced toxicity in rat. Obviously, a significant increase in tooth fluoride concentration was observed in the rats with fluorosis as shown in Fig. 2B. As expected, fluoride concentration of teeth in the fluorosis group and low‐, medium‐ and high‐dose gastrodin groups was higher than that in the control group, and some were statistically significant (*P* < 0.05).

The changes in fluorosis in rat incisors were divided into three grades. To standardize the study, authors followed the grading criteria described by Siddiqui [22]: normal, teeth are brownish yellow and shiny; mild, the teeth appear as transparent strips, with the teeth gradually faded brown‐yellow, showing a white jade‐like appearance; moderate, the surface of the teeth is transparent and banded, white plaque appears, and the enamel is lusterless and chalky; severe, besides the well‐established brown line, the surface has spot defect and enamel peeling off. The fluorosis conditions of the rats in each group in each time period were classified as shown in Table 1 and Fig. 2C. Except for the control group, all rats in each group demonstrated dental fluorosis at T1, with yellow pigment fading on enamel surface, enamel opacification and white stripes distributed along the transverse stripes. At T4, rats in the fluorosis group manifested moderate dental fluorosis with brown and white stripes. The condition of dental fluorosis in each gastrodin group eased and changed to mild‐to‐moderate dental fluorosis. According to Fig. 2D, gastrodin also improved fluoride‐induced dentinal tubule mineralization.

**Table 1:** Gastrodin alleviates the occurrence of dental fluorosis in rats.

Group	T	Normal	Mild	Moderate	Severe
Control	T1	12	0	0	0
	T2	12	0	0	0
	T3	8	0	0	0
	T4	4	0	0	0
Gastrodin	T1	12	0	0	0
	T2	12	0	0	0
	T3	8	0	0	0
	T4	4	0	0	0
Fluorosis	T1	3	8	1	0
	T2	0	6	6	0
	T3	0	4	3	1
	T4	0	0	2	2
Low‐dose gastrodin	T1	2	10	0	0
	T2	0	10	2	0
	T3	0	6	2	0
	T4	0	0	4	0
Medium‐dose gastrodin	T1	2	10	0	0
	T2	1	8	3	0
	T3	1	6	1	0
	T4	0	2	2	0
High‐dose gastrodin	T1	2	10	0	0
	T2	2	8	2	0
	T3	1	6	1	0
	T4	0	3	1	0

### Gastrodin reduced fluoride concentration of bone and inhibited oxidative damage in the serum and bone in rats

**Fig. 3:**
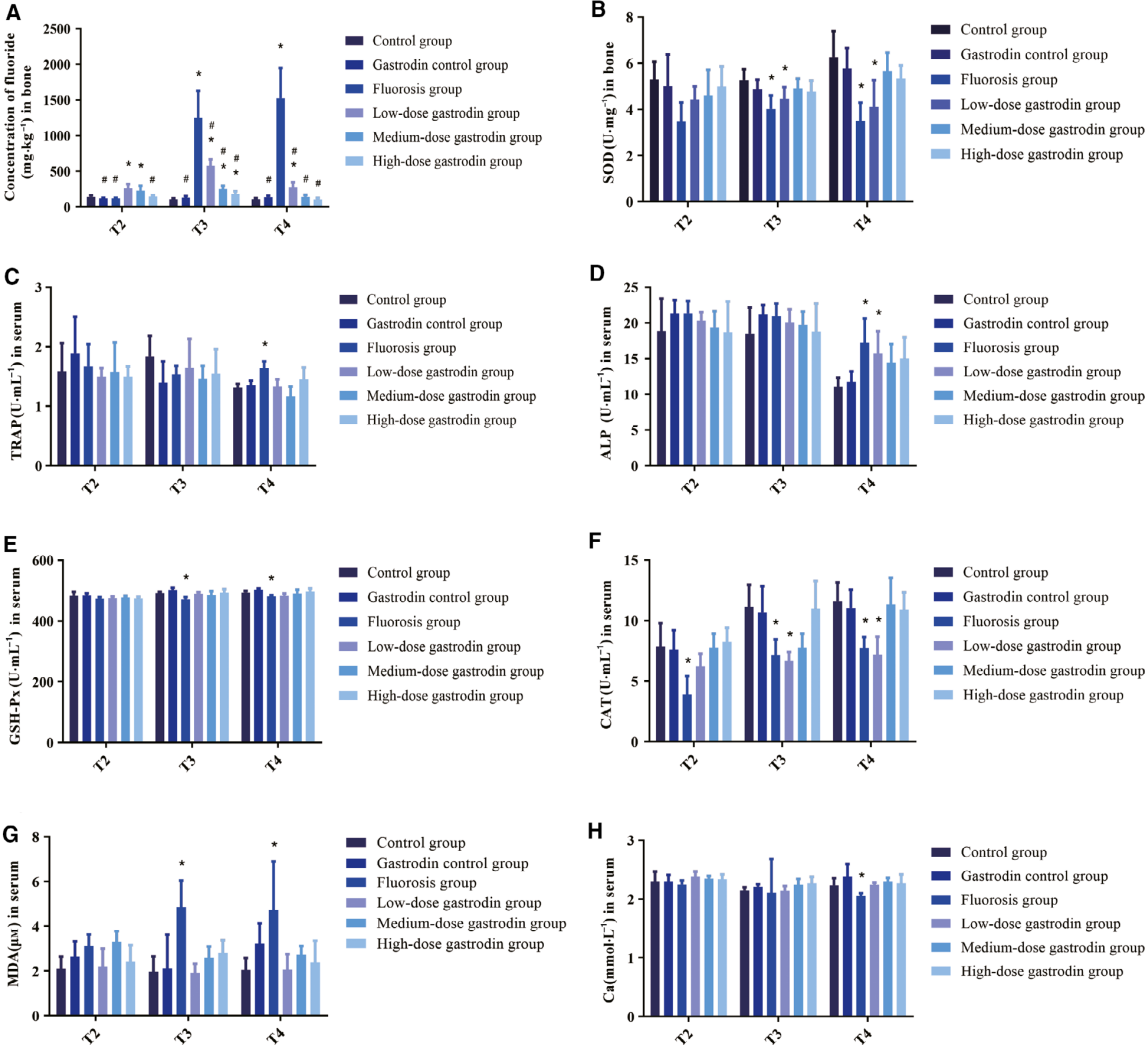
Fluoride concentration of bone tissue, serum ALP and TRAP activity and levels of oxidative stress‐related indicators among six groups (*n* = 4). (A) Bone fluoride concentration among six groups. (B) Bone tissue SOD activity among six groups. (C) Serum TRAP activity in each group. (D) Serum ALP activity of each group during the experiment. (E) Serum GSH‐Px activity in each group. (F) Serum CAT activity of each group. (G) Serum MDA activity in each group. (H) Serum Ca concentration of each group during the experiment. Data are expressed as the mean ± SD (*n* = 4). Data were analyzed by one‐way ANOVA with Bonferroni's test for multiple comparisons (A–H). *P < 0.05 versus control group; ^#^ *P* < 0.05 versus fluorosis group.

Obviously, fluoride concentration of bone in the fluorosis group and low‐, medium‐ and high‐dose gastrodin groups was higher than that in the control group (*P* < 0.05). Fluoride concentration of bone in the fluorosis group was significantly higher than in any other group (*P* < 0.05). Compared among the gastrodin groups, the bone fluoride decreased gradually with the increase of the dose of gastrodin, and there were differences in the fluoride concentration among the high‐, low‐ and medium‐dose gastrodin groups. This result would ensure that the treatment with fluoride has certain action in the bone, produces a stimulus of the bone tissue and an increase in the incorporation of fluoride, and various doses of gastrodin can reduce the fluorine concentration in the tissues accordingly (Fig. 3A).

To assess the protective mechanism of gastrodin on bone damage after fluorosis in rats, we analyzed the representative indicators of serum oxidative stress levels, markers of osteoclast activity and markers of osteoblast activity. The effects of gastrodin on the levels of GSH‐Px, Ca, CAT, TRAP, ALP, SOD and MDA in rats were shown in Fig. 3B–G. The serum GSH‐Px activity in the fluorosis group was lower than that in the control group, the SOD activity in the bone tissue of the fluorosis group was lower than that in the control group, and all were statistically significant (*P* < 0.05). Gastrodin also significantly attenuated the increased MDA level caused by fluoride, and the serum MDA concentration in the fluorosis group was higher than that in the control group (*P* < 0.05). The serum CAT activity of the rats in the fluorosis group was lower than that of the control group, and gastrodin also significantly increased the decreased CAT level caused by fluoride.

As shown in Fig. 3H, compared with the control group, the serum Ca concentration of the fluorosis group decreased; however, there was no significant difference. Compared with the control group, the serum ALP activity of the fluorosis group was increased, and at T2, the control group was significantly different from the fluorosis group and low‐ and high‐dose gastrodin groups (*P* < 0.05). Serum TRAP activity in the fluorosis group was increased only at T4 compared with the control group, and the difference was significant (*P* < 0.05).

### Antitrabecular bone pathological damage effects of gastrodin in rats with fluorosis

**Fig. 4:**
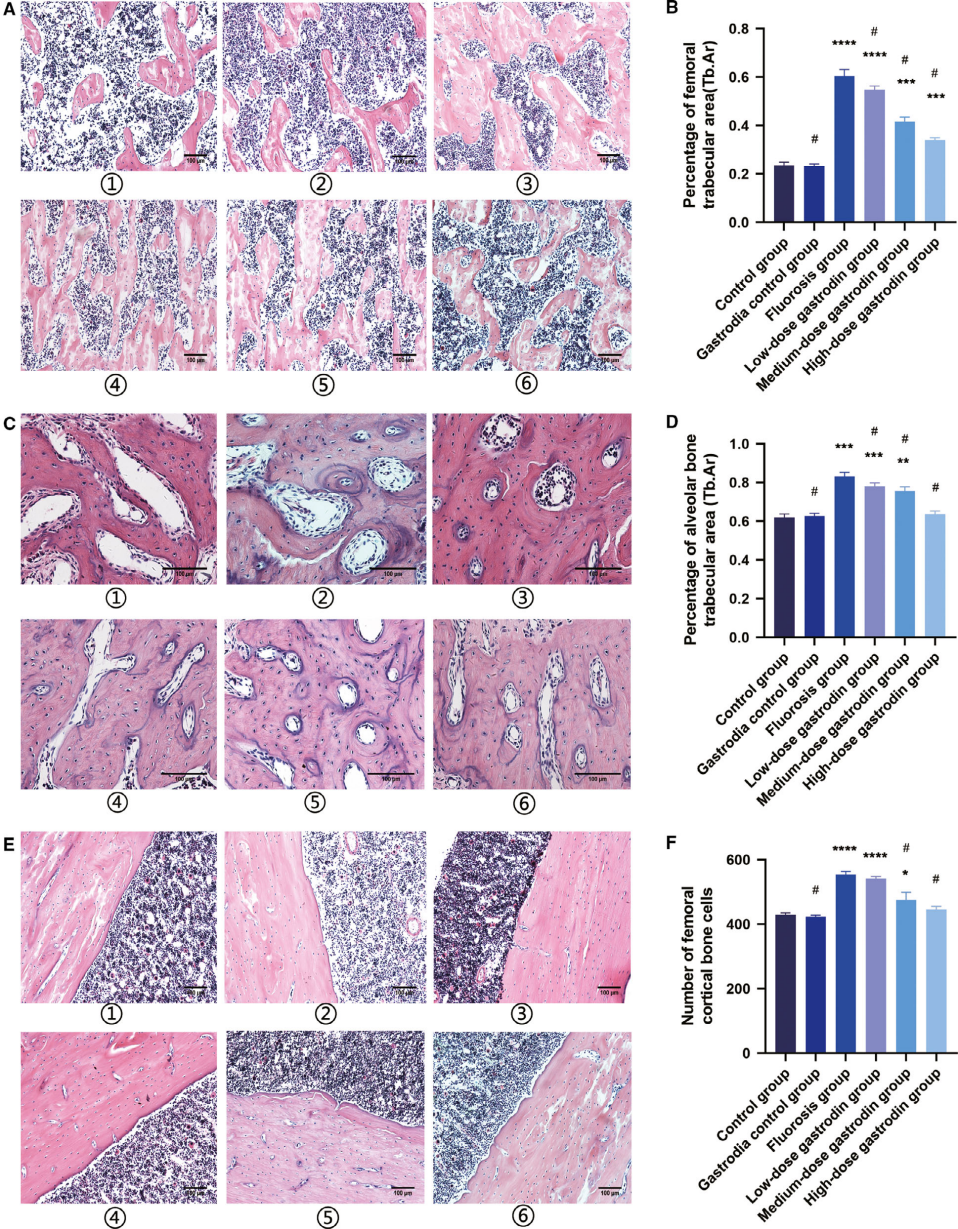
Gastrodin inhibits alveolar bone and femoral lesions in fluorosis in rats and does not show significant systemic toxicity. After fluoride intake for 30 days, rats were administered gastrodin for 90 days during treatment with fluoride. (A) The morphology of the cancellous bone of the femoral trabecular in each group during the experiment. (B) The analysis of percentage of femoral trabecular area. (C) The alveolar bone trabecular morphology of each group during the experiment. (D) The analysis of percentage of alveolar bone trabecular area. (E) The morphology of the femoral cancellous bone of each group during the experiment. (F) The analysis of number of femoral cortical bone cells. 2: Representative photomicrographs of sections of H&E‐stained organs. After treatment with gastrodin, no obvious abnormal changes were observed in the bone tissue sections, and no obvious toxicity was observed. 3: Representative micrograph of H&E‐stained skeletal fluorosis. The sample showed a marked area of abnormal bone hyperplasia. 6: Representative photomicrographs of H&E‐stained organ sections. After treatment with gastrodin, no obvious abnormal changes were observed in the tissue sections. (A, E) Original magnification, ×100; scale bars: 100 μm. (C) Original magnification, ×200; scale bars: 100 μm. 1: The control group; 2: the gastrodin control group; 3: fluoride; 4: fluoride + gastrodin (100 mg·kg^−1^); 5: fluoride + gastrodin (200 mg·kg^−1^); 6: fluoride + gastrodin (400 mg·kg^−1^). Data are expressed as the mean ± SD (*n* = 8). Data were analyzed by one‐way ANOVA with Bonferroni’s test for multiple comparisons (B, D, F). *P < 0.05 versus control group; **P < 0.01 versus control group; ***P < 0.001 versus control group; ****P < 0.0001 versus control group; ^#^ *P* < 0.05 versus fluorosis group.

To evaluate the effects of gastrodin on the trabecular bone microarchitectures, we administered different concentrations of gastrodin to rats. At 12 weeks, the rats were sacrificed. Change of the trabecular bone of the femur and alveolar bone demonstrated that fluoride deteriorated the trabecular bone microarchitectures, and as evaluated by the disordered trabecular bone, the thickness of the trabecular bone was thickened, the spacing became smaller, and the number of femoral osteocytes, percentage of alveolar bone trabecular area and percentage of femoral trabecular area increased compared with the control group (Fig. 4A–D). However, treatment of fluorosis in rats with gastrodin partially ameliorated these adverse effects and improved trabecular microarchitectures; the trabecular bone was evenly distributed, and the number of cells was obviously restored (Fig. 4E,F).

### Gastrodin inhibited cells apoptosis of bone tissue *in vivo*

**Fig. 5:**
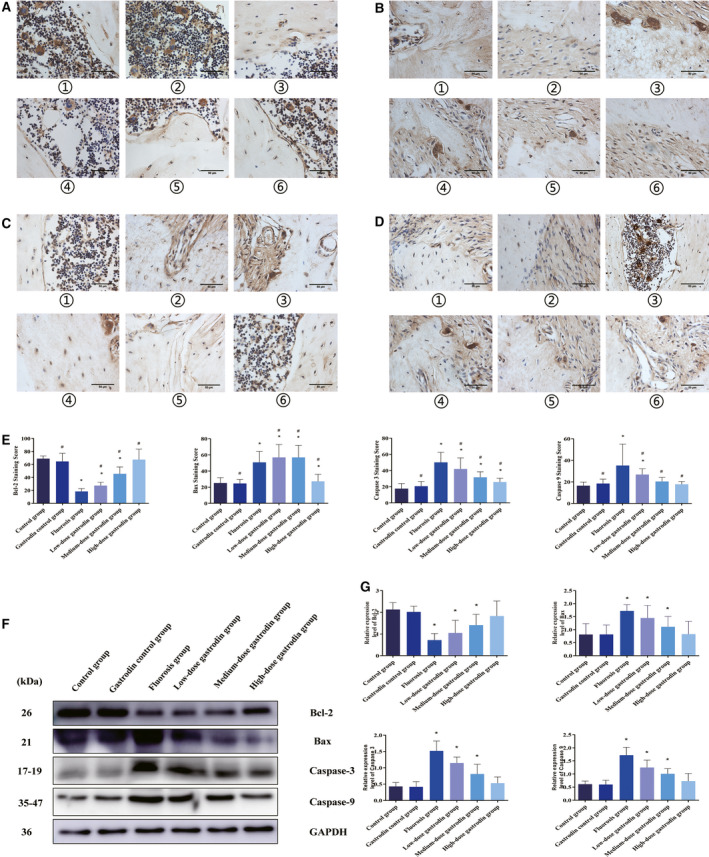
Gastrodin displays protective effects on chronic fluorosis and bone damage in the presence of fluoride (*n* = 10). After fluoride intake for 30 days, rats were administered gastrodin for 90 days during treatment with fluoride. Original magnification, ×400. (A–E) Effect and analysis of Bcl‐2, Bax, caspase‐3 and caspase‐9 protein expression in rat bone tissue by immunohistochemistry. (F, G) Analysis of Bcl‐2, Bax, caspase‐3 and caspase‐9 protein expression in rat bone tissue by western blot in the presence of fluoride, respectively. 1: The control group; 2: the gastrodin control group; 3: fluoride; 4: fluoride + gastrodin (100 mg·kg^−1^); 5: fluoride + gastrodin (200 mg·kg^−1^); 6: fluoride + gastrodin (400 mg·kg^−1^). (A, C, D, F) Original magnification, ×400; scale bars: 50 μm. Data are expressed as the mean ± SD (E, *n* = 8; G, *n* = 4). Data were analyzed by one‐way ANOVA with Bonferroni's test for multiple comparisons (E, G). *P < 0.05 versus control group; ^#^ *P* < 0.05 versus fluorosis group.

To examine the effect of gastrodin on bone tissue apoptosis in fluorosis rats, we measured the expression of apoptotic gene proteins, including caspase‐3, caspase‐9, Bax and Bcl‐2. Compared with the control group, all apoptosis markers, such as caspase‐3, caspase‐9 and Bax (Fig. 5B–E), were significantly increased after fluoride treatment. Compared with the control group, Bcl‐2 was significantly decreased after fluoride treatment (Fig. 5A). However, after treatment with gastrodin (100, 200, 400 mg·kg^−1^), tissue caspase‐3, caspase‐9 and Bax expression (Fig. 5B–E) were found to be significantly reduced. Gastrodin pretreatment significantly increased the expression of antiapoptotic protein compared with the fluoride‐treated group alone (Fig. 5F,G). Our data indicate that gastrodin reduces the fluoride‐induced bone structure lesions in rats.

### Gastrodin inhibited MC3T3‐E1 cell apoptosis induced by fluoride *in vitro*

**Fig. 6:**
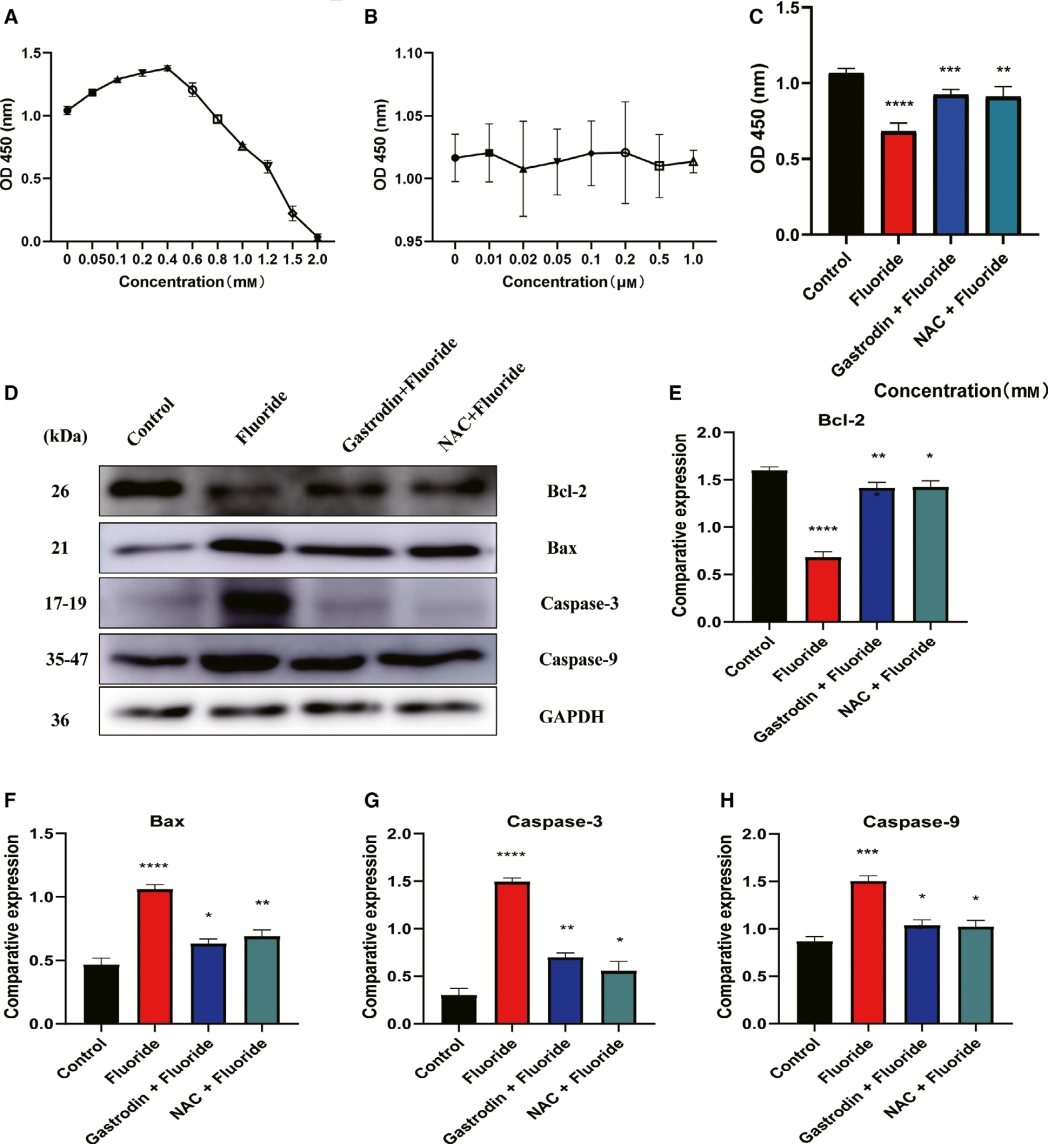
Gastrodin inhibited MC3T3‐E1 cell apoptosis induced by fluoride. (A) Effects of different concentrations of fluoride on the proliferation of MC3T3‐E1 cells. (B) Effects of different concentrations of gastrodin on the proliferation of MC3T3‐E1 cells. (C) Effects of gastrodin and NAC on the proliferation of MC3T3‐E1 cells treated with fluoride. (D–H) Effect and analysis of Bcl‐2, Bax, caspase‐3 and caspase‐9 protein expression in MC3T3‐E1 cell by western blot in the presence of fluoride, respectively. Data are expressed as mean ± SD (*n* = 3). Data were analyzed by one‐way ANOVA with Bonferroni's test for multiple comparisons (C, E–H). *P < 0.05 versus control group; **P < 0.01 versus control group; ***P < 0.001 versus control group; ****P < 0.0001 versus control group.

To examine the effect of gastrodin on MC3T3‐E1 cell apoptosis induced by fluoride, we measured the effect of gastrodin on inhibition of MC3T3‐E1 cell proliferation by fluoride. First, we determined that fluoride has a dual effect on osteoblasts. Low dose of fluoride (≤0.4 mm) promotes cell proliferation, as shown in Fig. 6A. In this experiment, a fluorine concentration of 0.6 mm was selected. As shown in Fig. 6B, different doses of gastrodin had no obvious inhibitory effect on cell proliferation. The concentration of gastrodin of 0.1 mm was selected and treated with ROS scavenger *N*‐acetyl‐l‐cysteine (NAC) as a positive control. Figure 6C shows that both gastrodin and NAC significantly reduced the proliferation inhibitory effect of fluorine on MC3T3‐E1 cells. As shown in Fig. 6D–H, all apoptosis markers, such as caspase‐3, caspase‐9 and Bax (Fig. 6), were significantly increased after fluoride treatment. However, after treatment with gastrodin (0.1 mm), caspase‐3, caspase‐9 and Bax expression (Fig. 6D–H) were found to be significantly reduced. Gastrodin pretreatment significantly increased the expression of antiapoptotic protein compared with the fluoride‐treated group alone.

## Discussion

Excessive intake of fluoride can lead to fluorosis, which is characterized by dental fluorosis and skeletal fluorosis. It is well known that the mechanism of fluorosis is the increasing of ROS levels in the body, including serum and bone tissue. ROS is mainly derived from mitochondrial activity, can damage various macromolecules and can consequently cause cell death and a series of pathological changes in bone tissue [6, 23, 24]. Previous work has documented that some kind of herb medicine could treat fluorosis in hard and soft tissues, as well as effectively alleviate the disturbance of redox balance in patients with fluorosis. Research has found that NaF significantly reduced the number of osteoclasts in rat bone tissue and slowed the movement of orthodontic teeth [25]. Gastrodin, a natural bioactive extract from the traditional Chinese herbal agent Gastrodia elata, can effectively remove oxygen free radicals and exert antioxidant activity in many ways [19]. This work found that *in vivo* NaF exposure induced fluoride accumulation in teeth and induced oxidative stress and activated caspase‐related pathway, and gastrodin treatment attenuated fluoride accumulation, reversed oxidative stress and inhibited the caspase‐related pathway, thus preventing apoptosis of the bone tissue, as demonstrated in Fig. 5.

In the past decade, the antioxidant activity of gastrodin has received extensive attention [26]. However, it has not been studied whether gastrodin has a protective effect on fluorosis injury. Several antioxidant and anti‐inflammatory agents, such as lycopene and fisetin, are effective to relieve oxidative stress caused by fluorosis, thereby playing a role in bone protection [27, 28]. Recently, a group of studies have manifested that gastrodin can scavenge free radicals and protect bone cells from oxidative stress [11, 29]. Our data have shown that gastrodin has a strong protective effect on fluoride‐mediated oxidative damage. Compared with the control group, 400 mg·kg^−1^ gastrodin had no significant negative effect on bone tissue, and the addition of gastrodin reduced the fluorine‐induced cytotoxicity. Different doses of gastrodin groups also inhibited free radical attack caused by fluorosis and protected the rats from oxidative damage. We also found that both gastrodin and NAC significantly reduced the proliferation inhibitory effect of fluorine on MC3T3‐E1 cells *in vitro*.

Animal studies have found that fluoride concentration in experimental animals increased with the increasing of fluoride concentration in drinking water. Błaszczyk *et al*. [30] found that rat treatment with NaF (10 mg·kg^−1^ of body mass/day) in drinking water generated obvious fluoride accumulation in teeth, which was similar with our results. Compared with the control group, rats subjected to long‐term exposure to NaF in drinking water had significantly elevated NaF concentrations in bone and teeth and decreased body weight. These results are consistent with the data from Weishan Li's group [10] that 10 mg·kg^−1^ concentration levels of fluoride drastically increased the concentration of fluoride in the NaF‐treated group significantly. The administration of gastrodin has significantly rescued the damage caused by fluoride according to the earlier indexes. When gastrodin is added 1 month after fluoride initiation, gastrodin strongly inhibits the accumulation of fluoride in the teeth and bones (Fig. 3). Similar to gastrodin, antioxidant components of various traditional Chinese medicine prescriptions can antagonize the oxidative stress injury induced by fluorosis, effectively prevent and reduce various organ damage caused by chronic fluorosis, and promote the excretion of fluorine [31]. However, although the fluorosis of the gastrodin group was weakened, it still existed in the later stage. This indicated that gastrodin might have a certain mitigating effect on fluorosis.

The growth and metabolism of bone tissue involves cell proliferation, activation and fusion. *In vitro* studies have stated that fluoride could inhibit the differentiation and maturation of osteoclasts and reduce bone resorption [1, 32]. A previous study found that ALP and TRAP play a paramount role in the bone metabolism of fluorosis, and fluoride can promote the increase of serum ALP activity, which is consistent with our findings [33]. The activities of ALP and TRAP were increased in rats with fluorosis; the serum ALP activity in the fluorosis group at T2 and the TRAP activity in the fluorosis group at T4 were significantly higher than that of the control group (*P* < 0.05), and medium and high doses of gastrodin attenuated the increase of fluoride ALP and TRAP in the rat body (Fig. 5). Elevated ALP activity may be one of the manifestations of bone metabolism disorder in skeletal fluorosis. In this experiment, a low dose of fluoride (≤0.4 mm) promoted cell proliferation, a high dose of fluoride (≥ 0.6 m) inhibited cell proliferation, and gastrodin inhibited MC3T3‐E1 cell apoptosis induced by fluoride.

Further histological observations showed effects of NaF treatment on histopathological changes and damaged structure in the bone of rats. Significant improvement in bone damage was observed after 3 months of ingestion of gastrodin and NaF. Interesting was that gastrodin also improved fluoride‐induced dentinal tubule mineralization.

Bartlett *et al*. [34] reported for the first time that increased cell apoptosis may play a role in the mechanism of fluorosis. Later, Singh *et al*. [35] demonstrated that fluoride suppresses proinflammatory cytokines expression, and thus affects DNA repair machinery with proapoptotic implications to trigger apoptosis. Also, Li *et al*. [10] proposed that fluoride‐induced ameloblastic apoptosis by oxidative stress resulted from reduction of enzymatic antioxidant. Elevated levels of Bcl‐2 and a decrease in Bax indicate that cells are more resistant to apoptosis and should be a marker of protective drug action, and vice versa. Studies have found that oxidative stress caused by high doses of NaF leads to mitochondrial damage, leading to osteogenic and osteoclast apoptosis [32, 36]. The caspase cascade is triggered by caspase‐9, after which caspase‐3 becomes activated by caspase‐9. Caspase‐3 degrades a variety of cytoplasmic and nuclear proteins and activates nucleases, thereby inducing DNA degradation [37, 38]. In this study, fluoride‐induced ROS were a harmful by‐product of mitochondrial respiration and activated caspase‐3 apoptosis pathway under stress conditions; it was proved that the protein levels of Bcl‐2 were down‐regulated, whereas caspase‐3, Bax and caspase‐9 were up‐regulated, in the fluorosis group, with cell death in this group significantly increased in *in vivo* and *in vitro* experiments. This results in reduced protection against cell death, leading us to infer that excessive fluoride induced cell death of bone tissue, whereas gastrodin administration showed significant functional recovery and morphological changes 3 months after treatment and Bcl‐2 expression was up‐regulated, whereas caspase‐3, Bax and caspase‐9 expression were down‐regulated, thereby enhancing the protective defense mechanism of fluoride‐induced rat body through antioxidant and anti‐inflammatory pathways.

In conclusion, gastrodin may reduce the level of ROS, improve the trabecular microstructure of rats with fluorosis and alleviate the toxic effects of fluoride on bone tissue by regulating the expression of Bcl‐2, Bax, caspase‐3 and caspase‐9 proteins. Gastrodin at 400 mg·kg^−1^ is found to be the optimum concentration against fluorosis. It might be plausible to expect gastrodin to be a promising therapeutic agent targeting fluoride‐induced oxidative stress and bone damage in the body caused by skeletal fluorosis. However, the processes are complicated; other pathways are possibly involved and should be further studied. Also, this effect and the optimal dose of gastrodin administration require further exploration.

## Conclusions

Gastrodin can provide a markedly protective effect against NaF‐induced bone tissue apoptosis and dental fluorosis by decreasing oxidative stress and down‐regulating the caspase pathway, indicating that gastrodin might act as a promising safe and effective agent for treatment of dental fluorosis in rats. Although further studies are required, we can still propose gastrodin for the rescue from fluoride toxicity.

## Conflict of interest

The authors declare no conflict of interest.

## Author contributions

FKM wrote the manuscript. BZ did the main experiments. CS collected and analyzed the raw data. JH and AOA helped to revise the article. YL designed the whole work. All authors read and approved the final manuscript.

## Data Availability

The additional data used to arrive at these conclusions can be obtained from the corresponding author on reasonable request.

## Data Accessibility

The additional data used to arrive at these conclusions can be obtained from the corresponding author on reasonable request.
